# Genomic analyses of multidrug resistant *Pseudomonas aeruginosa* PA1 resequenced by single-molecule real-time sequencing

**DOI:** 10.1042/BSR20160282

**Published:** 2016-11-29

**Authors:** Gang Li, Mengyu Shen, Shuai Le, Yinling Tan, Ming Li, Xia Zhao, Wei Shen, Yuhui Yang, Jing Wang, Hongbin Zhu, Shu Li, Xiancai Rao, Fuquan Hu, Shuguang Lu

**Affiliations:** * Department of Microbiology, College of Basic Medical Sciences, Third Military Medical University, Chongqing 400038, China

**Keywords:** genomic analyses, multidrug resistance, *Pseudomonas aeruginosa*, resequencing, single-molecule real-time technology

## Abstract

As a third-generation sequencing (TGS) method, single-molecule real-time (SMRT) technology provides long read length, and it is well suited for resequencing projects and *de novo* assembly. In the present study, *Pseudomonas aeruginosa* PA1 was characterized and resequenced using SMRT technology. PA1 was also subjected to genomic, comparative and pan-genomic analyses. The multidrug resistant strain PA1 possesses a 6,498,072 bp genome and a sequence type of ST-782. The genome of PA1 was also visualized, and the results revealed the details of general genome annotations, virulence factors, regulatory proteins (RPs), secretion system proteins, type II toxin–antitoxin (T–A) pairs and genomic islands. Whole genome comparison analysis suggested that PA1 exhibits similarity to other *P. aeruginosa* strains but differs in terms of horizontal gene transfer (HGT) regions, such as prophages and genomic islands. Phylogenetic analyses based on 16S rRNA sequences demonstrated that PA1 is closely related to PAO1, and *P. aeruginosa* strains can be divided into two main groups. The pan-genome of *P. aeruginosa* consists of a core genome of approximately 4,000 genes and an accessory genome of at least 6,600 genes. The present study presented a detailed, visualized and comparative analysis of the PA1 genome, to enhance our understanding of this notorious pathogen.

## INTRODUCTION

*Pseudomonas aeruginosa* is a glucose non-fermentative Gram-negative bacillus that can adapt to various ecological niches, such as soil, marshes, coastal marine habitats, and plant and animal tissues [1,2]. As an opportunistic pathogen, *P. aeruginosa* causes a wide range of syndromes in humans; in some instances, its infection is fatal and thus considered an increasingly notorious pathogen in nosocomial infection [3]. *P. aeruginosa* is significantly associated with respiratory tract infections, burn infections and urinary-tract infections in catheterized patients [4]. It is also the dominant pathogen in cystic fibrosis (CF) lung disease, with single lineage persisting throughout the whole life of a patient [5]. In infections among patients with CF, endocarditis and periodontitis, *P. aeruginosa* can form biofilms. *P. aeruginosa* can resist numerous antibiotics because of intrinsic drug resistance. The prevention and treatment of *P. aeruginosa* are very difficult because of biofilm formation and drug resistance [6,7].

Whole genome sequencing is commonly applied to analyse and understand the genotype and phenotype of an organism. Twenty-seven complete genome sequences of *P. aeruginosa* have become available in GenBank [8] since November 20, 2015, when our study was in progress. The completion of hundreds of genome scaffolds or contigs of *P. aeruginosa* is also underway. Some databases have been established, such as *Pseudomonas* Genome DB [9] (http://www.pseudomonas.com/), and they are very useful for analysis of *P. aeruginosa*. In the past less than two decades, the genomic, evolutionary and diversity studies of several *P. aeruginosa* strains have been performed [2,5,9–13]. However, many *P. aeruginosa* genome sequences and their corresponding annotations are still text files without a detailed and visualized genomic analysis. Similar to the genomes of other bacterial species, *P. aeruginosa* genomes share significant similarity, although they have been isolated from different niches or clinical origins [14,15]. Therefore, these similar genomes can form a genomic pool known as a pan-genome [16,17], and thus provide insights into virulence, drug resistance and biofilm formation related to the pathogenicity of *P. aeruginosa*.

*P. aeruginosa* strain PA1 was originally isolated from a patient with respiratory tract infection at the Second Affiliated Hospital of the Third Military Medical University; this strain belongs to serogroup 9 of *P. aeruginosa* international antigenic typing system [18,19]. In 2012, *P. aeruginosa* PA1 was sequenced by Solexa (Illumina). Unfortunately, more than 100 gaps have been generated when sequencing reads were assembled using the genome sequence of *P. aeruginosa* PAO1 as a reference. The final genome sequence of PA1 is 6,528,877 bp long [20]; nevertheless, we found that this genomic sequence of PA1 is not precise and thus should be resequenced. In the light of the third-generation sequencing (TGS) technologies represented by Pac-Bio single-molecule real-time (SMRT) sequencing that can provide long read lengths and high throughput to enhance *de novo* assembly, the PA1 genome was resequenced using SMRT technology in 2015. The present study presented a detailed and visualized genomic analysis of *P. aeruginosa* PA1 genome. The present study also performed a comparative, phylogenetic and pan-genomic analysis of *P. aeruginosa* genomes to provide a useful basis for future studies of this notorious pathogen.

## MATERIALS AND METHODS

### Bacterial growth and Gram staining

*P. aeruginosa* PA1 was isolated and stored in our laboratory [18,19]. The bacteria were grown in LB broth or plated on to a LB medium containing 1.5% (w/v) agar. Afterwards, 100 μl of log-phase PA1 liquid cultures were added to 100 ml of LB medium, and the mixture was incubated at 37°C with shaking at 220 rpm for 30 h. Then, 150 μl of liquid cultures were obtained at an interval of 1 h to examine the corresponding *A*_600_ by using a SmartSpecTM3000 spectrophotometer (Bio–Rad Laboratories) and to provide data for growth curve. Gram staining was performed as previously described [21].

### TEM

The log-phase PA1 liquid cultures (approximately 10^8^ CFU/ml) were placed on copper grids to undergo adsorption for 10 min, negatively stained with 2% phosphotungstic acid (PTA, pH 4.5) for 15 s and air-dried. Bacterial samples were observed using a TECNAI 10 electron microscope (Philips) at a voltage of 80 kV and a magnification of 65,000. Images were acquired digitally with a camera (Gatan Model 785) inside the microscope.

### Minimal inhibitory concentration assay

Minimal inhibitory concentration (MIC) assay was performed at the Second Affiliated Hospital of the Third Military Medical University (Chongqing, China) by using a VITEK-2 Compact system with Advanced Expert System (bioMerieux) in accordance with the manufacturer's instructions. Twenty-nine antibiotics were evaluated, and MIC interpretive standards for *P. aeruginosa* were based on Performance Standards for Antimicrobial Susceptibility Testing (M100-S25, January 2015) (http://clsi.org/).

### DNA extraction and SMRT resequencing

The PA1 genomic DNA was extracted and purified from the stationary phase cultures grown in LB broth by using a TIANamp bacteria DNA kit (TIANGEN BIOTECH). Approximately 10 μg purified PA1 genomic DNA was then subjected to SMRT sequencing at the Institute of Medicinal Plant Development (IMPLAD, Beijing, China) by using PacBio RS (Pacific Biosciences) [22]. SMRTbell template libraries with DNA fragments of 5 kb were prepared. PA1 genomic DNA was fragmented using Covaris microTUBE (ThermoFisher Scientific) and then purified by AMPure PB Beads (http://www.pacb.com/products-and-services/consumables/pacbio-rs-ii-consumables/sample-and-template-preparation-kits/). Seque-ncing was then performed using four SMRT cells and zero-mode waveguide (ZMW) [23] signals were obtained. *De novo* assembly was performed by using RS_HGAP_Assembly v. 2.0 [24], and single contig with an average sequence coverage of 396.2-fold was revealed.

### Sequence analysis and genome annotation

DNAStar [25] and DNAMAN (http://www.lynnon.com/) were used to analyse the general features of the PA1 genome sequence. The PA1 genome, including genes, proteins, rRNAs and tRNAs, was annotated through the NCBI Prokaryotic Genome Automatic Annotation Pipeline (PGAAP) (http://www.ncbi.nlm.nih.gov/genome/annotation_prok/) [26]. Antibiotic resistance genes were predicted by the ResFinder-2.1 Server (https://cge.cbs.dtu.dk/services/ResFinder/) [27]. Prophages were predicted by PHAST (http://phast.wishartlab.com/) [28]. Restriction–modification (R–M) systems were predicted in REBASE (http://rebase.neb.com/rebase/rebase.html) [29]. PA1 was subjected to multilocus sequence typing by using MLST 1.8 online server (https://cge.cbs.dtu.dk/services/MLST-1.8/) [30]. MLST configuration was selected as “*P. aeruginosa*”, and type of the reads was selected as “Assembled Genomes/Contigs”. The complete genome sequence of *P. aeruginosa* PA1 with “.fasta” format was considered as input data. PathogenFinder 1.1 (https://cge.cbs.dtu.dk/services/PathogenFinder/) [31] was used to analyse the complete genome sequence of *P. aeruginosa* PA1. The phylum was chosen as “γ-proteobacteria”, and the Sequencing Platform was selected as “Assembled Genomes/Contigs”. The complete genome sequence of *P. aeruginosa* PA1 with “.fasta” format was also used as input data.

### Visualized analysis of the PA1 genome

The old and new genome sequences of PA1 were subjected to pairwise nucleotide sequence comparison in EasyFig (http://mjsull.github.io/Easyfig/) [32]. The PA1 genome was then circularly presented by using Basic Local Alignment Search Tool (BLAST) Ring Image Generator (BRIG) (http://brig.sourceforge.net/) [33] and CGView (http://stothard.afns.ualberta.ca/cgview_server/) [34]. BRIG and CGView results were combined to present the genome map. The virulence factors and secretion system proteins of PA1 were manually selected from the GenBank file of PA1 and visualized with BRIG. Regulatory proteins (RPs) were predicted by Predicted Prokaryotic Regulatory Proteins server (http://www.p2rp.org/) [35] and visualized using BRIG. Type II toxin–antitoxin (T–A) systems were predicted using TAfinder (http://202.120.12.133/TAfinder/index.php) [36] and visualized using BRIG. Genomic islands were analysed by IslandViewer (http://www.pathogenomics.sfu.ca/islandviewer/) [37].

### Comparative genomic analysis

The 27 complete *P. aeruginosa* genome sequences were compared through BlastN by using blast 2.2.29+ (ftp://ftp.ncbi.nlm.nih.gov/blast/) [38] and visualized by BRIG with 80% identity cut-off. The PA1 genome was used as reference. The 16S rRNA sequences of *Pseudomonas* sp. and four common bacterial strains were downloaded from *Pseudomonas* Genome Database (http://www.pseudomonas.com/) [39] and GenBank [8]. 16S rRNA sequences were subjected to multiple sequence alignments by using ClustalW [40] with default parameters, and phylogenetic trees were constructed and displayed by MEGA 6.06 (http://www.megasoftware.net/) [41] with the neighbour-joining method [42]. The topology is displayed. A Venn diagram was drawn by using EDGAR software platform (https://edgar.computational.bio.uni-giessen.de/cgi-bin/edgar_login.cgi?cookie_test=1) [43]. Pan-genome analysis was performed using EDGAR and Panseq (https://lfz.corefacility.ca/panseq/page/index.html) [44], with default parameters, and the results of EDGAR and Panseq were combined to present the pan-genome of *P. aeruginosa*.

## RESULTS

### Typical biological features of *P. aeruginosa* PA1

**Figure 1:**
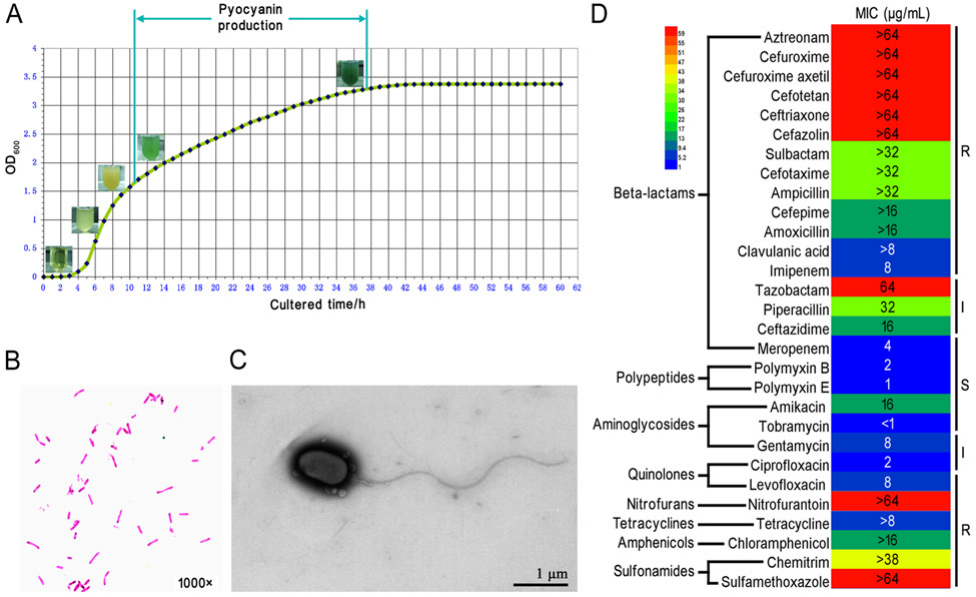
Growth, morphology and MIC properties of *P. aeruginosa* PA1 (**A**) Growth curve of *P. aeruginosa* PA1 growing in LB. The corresponding colour variation of bacterial liquid in different growing phases was shown in the test tube, and PCN production was indicated. (**B**) Gram staining of *P. aeruginosa* PA1. (**C**) TEM morphology of *P. aeruginosa* PA1. (**D**) MIC test of *P. aeruginosa* PA1. Twenty-nine antibiotics were tested, R: Resistant; I: Intermediate-resistant; S: Sensitive.

Pyocyanin (PCN) is a blue redox-active phenazine and secondary metabolite that contributes to the persistence of *P. aeruginosa* infections [45]. *P. aeruginosa* PA1 can produce apparent PCN after this strain is cultured in LB broth at 37°C for over 10 h. A growth curve with a straight line between log phase and stationary phase is then obtained (Figure 1A). Gram staining showed that PA1 is a Gram-negative rod-shaped bacterium with a heterogeneous length (Figure 1B). TEM revealed that PA1 secretes abundant extracellular matrix and some vesicles around the cellular surface, and most PA1 consists of one wave-like flagellum with a length of approximately 5 μm (Figure 1C), although a few strains contain two or three flagella.

### P. *aeruginosa* PA1 is a multidrug-resistant bacterium

Although *P. aeruginosa* species exhibits intrinsic drug resistance [46], the drug-resistant patterns of different strains remain distinct. *P. aeruginosa* PA1 was resistant or intermediate-resistant to most of the antibiotics tested (24 out of 29; Figure 1D), including 16 kinds of antibiotics that belong to β-lactams and 8 kinds of antibiotics that belong to nitrofurans, amphenicols, tetracyclines, sulfonamides, quinolones and aminoglycosides. However, only four antibiotic resistance genes were predicted in the PA1 genome (Supplementary Table S1). PA1 was sensitive to only five of the tested antibiotics, including polymyxin B and E that belong to polypeptides, amikacin and tobramycin that belong to aminoglycosides, and meropenem that belongs to β-lactams. *P. aeruginosa* PA1 is very difficult to prevent and treat because of multidrug resistance.

### Resequencing of the PA1 genome by SMRT method

**Figure 2:**
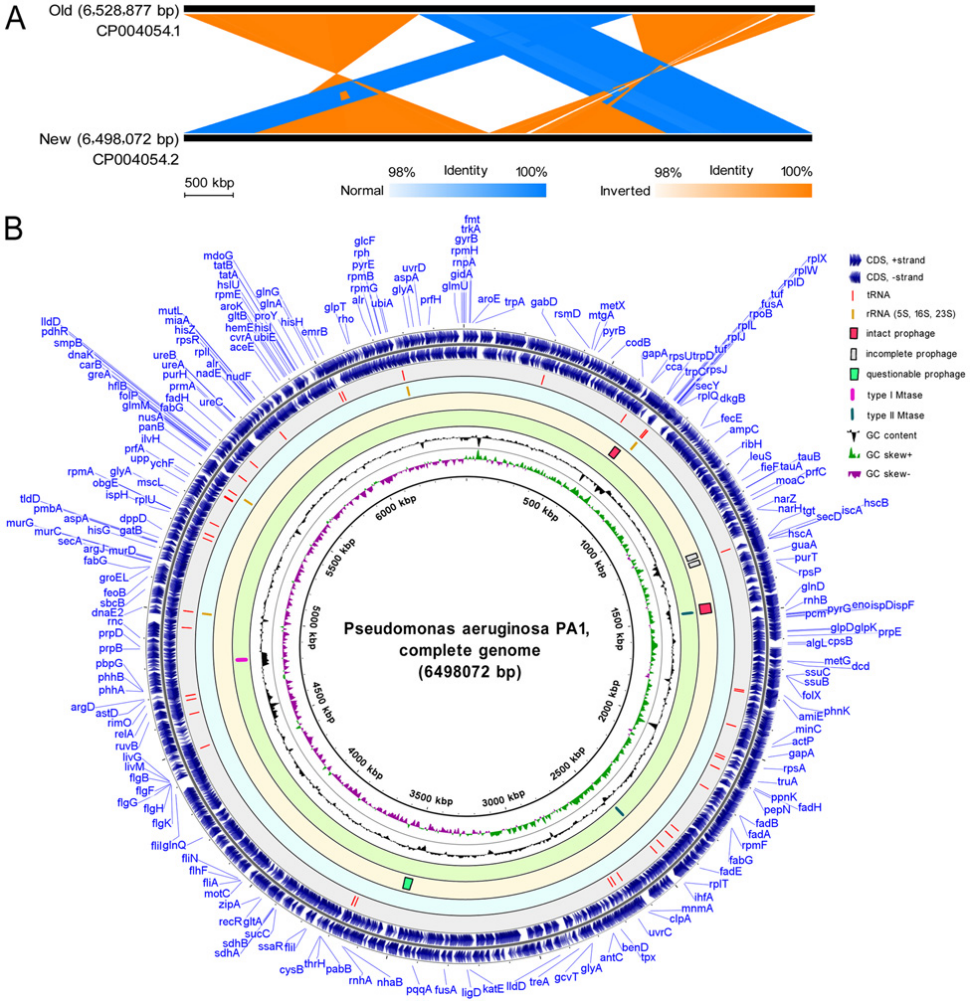
Resequencing and circular presentation of the *P. aeruginosa* PA1 genome (**A**) Pairwise nucleotide sequence comparison of the old and new genome sequences of PA1 (resequenced by SMRT technology). (**B**) Circular presentation of the PA1 genome. The names of 219 annotated genes (also shown in the GenBank file of PA1 genome) were indicated in blue in the outermost region. The outermost ring depicts the genes on the plus strand, followed by rings depicting the genes on the minus strand, tRNAs, rRNAs, predicted prophages, putative MTases, the GC content (black) and GC skew (purple/green).

The old genome sequence of *P. aeruginosa* PA1 (GenBank accession Number CP004054.1), containing 6,528,877 bp with 66.34% G+C content, was determined using Illumina Solexa with short reads (approximately 100 bp in length) and a mean coverage of 161× [20]. We found some point mutations of the old genome sequence as revealed by the results of PCR sequencing of the corresponding genome regions. This result indicated that the old sequence of PA1 lacks fidelity. However, the improved 6,498,072 bp complete genome sequence of PA1 (GenBank accession Number CP004054.2), resequenced by using SMRT technology [47,48], is accurate, which was validated by PCR sequencing. Compared with the new genome sequence of PA1, the old sequence contains many errors, such as nucleotide sequence mutations, inverted regions and contig arrangement errors (Figure 2A).

### Visualized analysis of *P. aeruginosa* PA1 genome

#### General features of the PA1 genome

The *de novo* assembly of the PA1 genome revealed single contig with a 396-fold sequence coverage, and the completed PA1 genome yields a G+C content of 66.35%. The general features of the PA1 genome are listed in Supplementary Table S2. Of the 5,902 predicted proteins, 78.43% exhibit putative functions. The circular genome map is shown in Figure 2(B). The PA1 genome carries four putative prophages and encodes one type I DNA methyltransferase (MTase) and two type II DNA MTases (Figure 2B). Multilocus sequence typing analysis [30] revealed that the sequence type of *P. aeruginosa* PA1 is ST-782, with a MLST profile as “*paeruginosa*”. The detailed matches are listed in Supplementary Table S3. No gaps were found within the tested gene locus. Using PathogenFinder [31], we predicted that the probability of *P. aeruginosa* PA1 as a human pathogen is 88.5%. The detailed results are listed in Supplementary Table S4. A total of 406 pathogenic families were matched with the complete genome sequence of *P. aeruginosa* PA1.

#### Virulence factors

**Figure 3:**
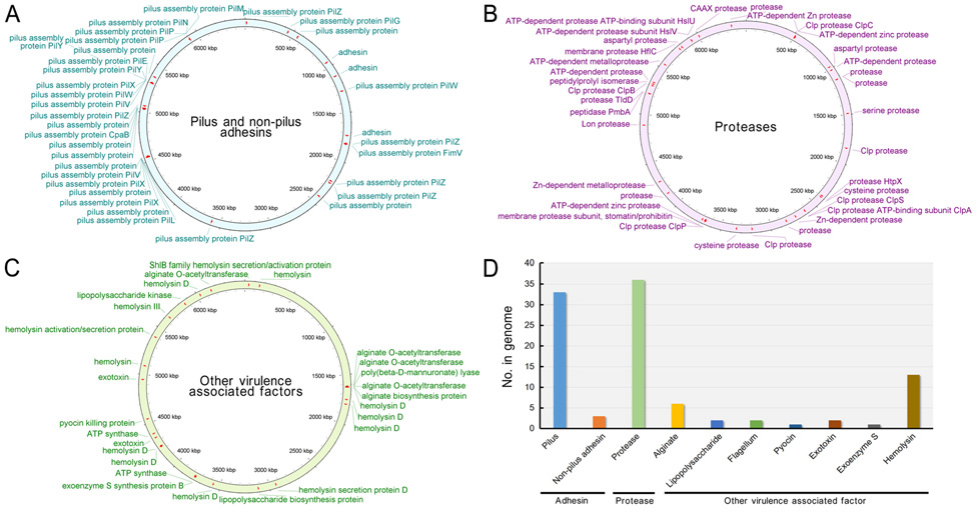
Virulence factors of *P. aeruginosa* PA1 (**A**) Distribution of pilus and non-pilus adhesins in the PA1 genome. (**B**) Distribution of proteases in the PA1 genome. (**C**) Distribution of other virulence-associated factors in the PA1 genome. (**D**) Counts of the virulence factors encoded by PA1.

The virulence factors of *P. aeruginosa* play an important role in the pathogenesis of *P. aeruginosa-*induced infections, such as keratitis, burn wound infections and respiratory tract infections. These factors include secretory virulence factors, such as protease, elastase, phospholipase, PCN, exotoxin A, exoenzyme S, haemolysins and siderophores or cell-associated factors, such as lipopolysaccharide (LPS), alginate, flagellum, pilus and non-pilus adhesins [49]. *P. aeruginosa* PA1 encodes 36 adhesins, 36 proteases and 27 other virulence-associated factors, such as alginate, LPS, flagellum, pyocin, exotoxin, exoenzyme S and haemolysin (Figure 3). These virulence factors are evenly distributed in the PA1 genome. No elastase, phospholipase or siderophore was predicted in the PA1 genome. By comparison, the standard *P. aeruginosa* strain PAO1 genome encodes one elastase, six phospholipases and one siderophore. This may indicate distinct virulence characteristics of these two strains.

#### Regulatory proteins

**Figure 4:**
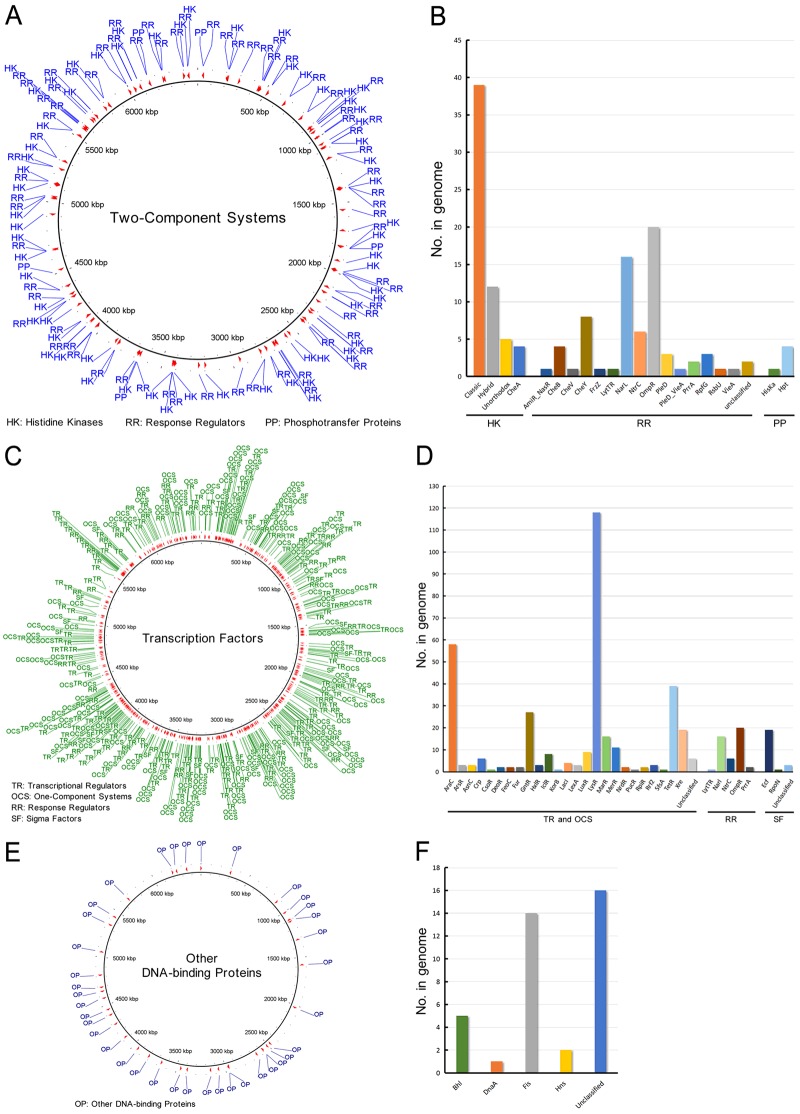
RPs of *P. aeruginosa* PA1 (**A**) Distribution of TCS in the PA1 genome. (**B**) Numbers and details of TCS proteins encoded by PA1. (**C**) Distribution of TFs in the PA1 genome. (**D**) Numbers and details of TFs encoded by PA1. (**E**) Distribution of ODP in the PA1 genome. (**F**) Numbers and details of ODP encoded by PA1.

RPs, such as transcription factors (TFs), two-component systems (TCS) and other DNA-binding proteins (ODP), are involved in the control of diverse cellular systems. RPs trigger bacterial adaptive responses to changes in environmental conditions [35]. As a versatile opportunistic pathogen, *P. aeruginosa* PA1 contains a set of RPs (Figure 4). The TCS of PA1 contains 63 histidine kinases, 71 response regulators and 5 phosphotransfer proteins (Figures 4A and 4B), which contribute to dominant phosphorylation-dependent signal transduction pathways of PA1. The TFs of PA1 comprise 175 transcriptional regulators, 175 one-component systems, 45 response regulators and 26 sigma factors (Figures 4C and 4D). PA1 also encodes 38 ODP, including Bhl, DnaA, Fis, Hns and 16 unclassified ODP (Figures 4E and 4F). The details of PA1 RPs are also listed in Supplementary material ‘Excel S1’. The number of PA1 RPs is different from other *P. aeruginosa* strains, e.g. PA1 has 139 TCS-related ORFs whereas PAO1 has 118 ones [2].

#### Secretion systems

**Figure 5:**
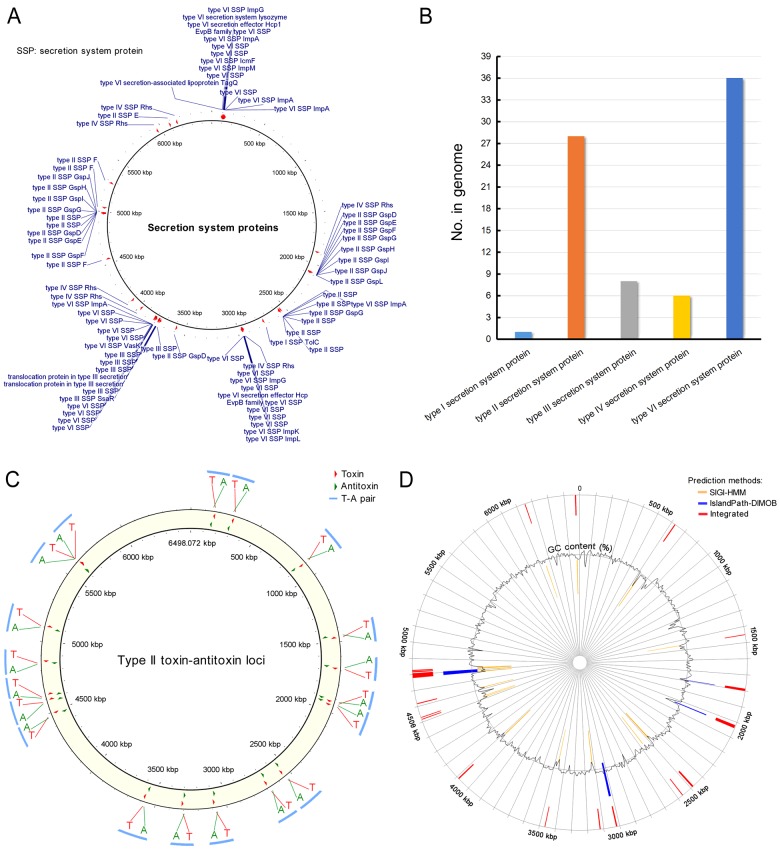
Secretion systems, type II T–A loci and genomic islands of *P. aeruginosa* PA1 (**A**) Distribution map of secretion proteins in the PA1 genome. (**B**) Counts of the secretion system proteins encoded in the PA1 genome. (**C**) Distribution of type II T–A loci in the PA1 genome. (**D**) Genomic islands in the PA1 genome.

Gram-negative bacteria consist of secretion systems from type I to type VIII, and secreted proteins, such as degradative enzymes or virulence factors, are the main tools that bacteria use to interact with their environment. With functional roles, secreted proteins provide potential therapeutic and commercial benefits [50]. *P. aeruginosa* PA1 encodes one type I, 28 type II, 8 type III, 6 type IV and 36 type VI secretion system proteins (Figure 5). Compared with PA1, PAO1 encodes only three type VI secretion system proteins and has no type I or type IV secretion system proteins. The distribution map of secretion proteins in the PA1 genome indicates that secretion proteins with the same type usually gather in a small genomic region (Figure 5A). One type I secretion protein (TolC) was found in the PA1 genome (Figure 5B), and TolC is an outer membrane protein involved in the export of chemically diverse molecules, including large protein toxins, such as α-haemolysin and small toxic compounds, such as antibiotics; thus, TolC is accounted for the virulence and multidrug resistance of pathogenic bacteria [51].

#### Type II toxin–antitoxin systems

T–A systems, as one of the simplest classes of genes involved in the programmed death of bacteria, are small genetic modules abundant in bacterial genomes [52]. T–A systems can be categorized into five types on the basis of the nature and mode of action of the antitoxin component (from type I to type V) [53]. Type II T–A systems are highly represented because of their ability to move by horizontal gene transfer (HGT). In type II T–A system, antitoxin and toxin are proteins, and they become neutralized by forming a T–A complex. The PA1 genome was predicted to have 19 type II T–A pairs (Figure 5C) on the basis of sequence alignment and conserved domain searches against the diverse T–A families. We predicted the type II T–A pairs of other three *P. aeruginosa* strains, PAO1, PA7 and PA14. The results suggested that the numbers of the type II T–A pairs of these three strains are 14, 18 and 17 respectively. The details of the predicted type II T–A proteins in the PA1 genome are listed in Supplementary Table S5. Among these pairs, a T–A pair is coded in a prophage region from 1,442 kb to 1,488 kb (Figure 2B). This T–A pair comprises an antitoxin (PA1S_06865) containing a RHH-like domain and a toxin (PA1S_06870) consisting of a COG2929-like domain. The T–A pair has been identified and characterized as a HicAB T–A System in *P. aeruginosa* by our group [54].

#### Genomic islands

Many bacterial genomes contain genomic islands that are mostly acquired and exchanged through lateral gene transfer [55]. Genomic islands typically differ in their G+C content and encode various accessory activities involved in unique functions, such as symbiotic and pathogenesis functions [56]. The PA1 genome was predicted to have 19 genomic islands spanning 138 genes (Figure 5D). The details of the contents of the predicted genomic islands of PA1 are shown in Supplementary material ‘Excel S2’. The average length of a PA1 genomic island is 8.9 kb, which is relatively large and thought to have horizontal origins. The largest genomic island of PA1 is 29.8 kb (from 4,778.5 kb to 4,808.3 kb, named as PA1_GI14) in length and is composed of 23 genes. The PA1_GI14 genomic island encodes proteins of a type I R–M system and an integrase and three transposases (Figure 2B). This R–M system is probably acquired by HGT. The genomic island PA1_GI2 (from 1,448.8 kb to 1,452.9 kb) is completely contained in a prophage region, and genomic island PA1_GI6 (from 2,563.5 kb to 2,568.6 kb) encodes four type II secretion system proteins. Compared with other genomic islands in *P. aeruginosa* strains described previously in literature [56–60], PA1_GI8 shares 99% identity with LESGI-4. A section of PA1_GI11, PA1_GI12, PA1_GI14 and PA1_GI15 shows over 90% identity with PAPI-2, PAPI-1, PAGI-8 and LESGI-1 respectively. Nevertheless, the details of PA1 genomic islands should be further investigated.

### Comparative genomic analysis of *P. aeruginosa*

#### Whole genome comparison analysis

**Figure 6:**
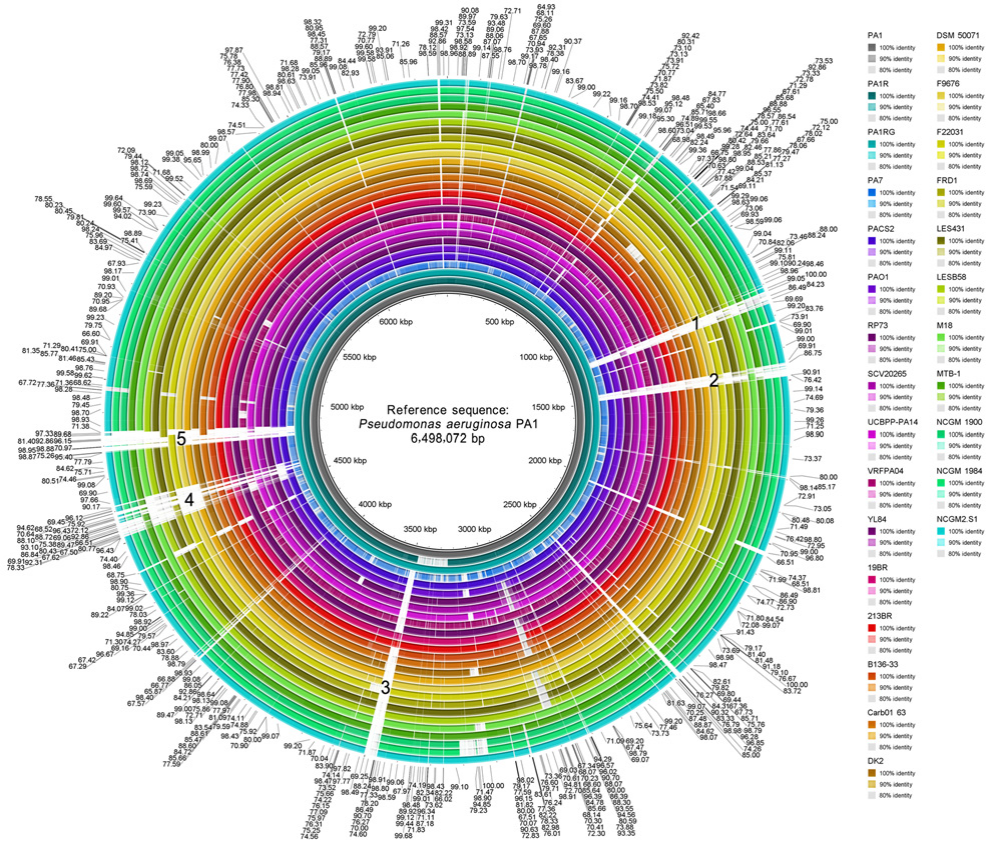
BLAST comparison of the complete genome of *P. aeruginosa* PA1 against 26 other *P. aeruginosa* strains through BRIG Identity labels of the 27 *P. aeruginosa* strains are shown in the same order as the rings from the innermost layer to the outermost layer. Identities of the corresponding comparison fragments are shown in the outermost region of this figure. Large low-identity genomic regions (over 30 kb in length) are indicated by numbers 1 to 5.

With the extensive improvement of next-generation sequencing (NGS) and TGS technologies over the last 10 years, publicly available complete bacteria genomic data have increased significantly [61]. Visualized genome comparison is necessary to help determine genotypic differences between closely related bacteria. As of November 20, 2015, 27 complete genome sequences of *P. aeruginosa*, including PA1, have been released from GenBank, and the detailed lists of the 27 complete *P. aeruginosa* genomes are shown in Supplementary Material ‘Excel S3’. The BRIG comparison [33] of these genomes showed that the genomic sequences of *P. aeruginosa* are highly similar, with the overwhelming majority of compared genomic regions revealing identity of over 80% against the PA1 genome (Figure 6). However, several relatively large genomic regions (over 30 kb in length) remain blank (with an identity less than 80%), as indicated by numbers 1 to 5 in Figure 6. The lengths of the regions numbered 1 to 5 are 38.7, 41.8, 39.6, 102.0 and 63.7 kb respectively. The five regions are contained in prophages (Figure 2B) or genomic islands (Figure 5D). This finding suggested that they are probably acquired by HGT during bacterial evolution.

#### Phylogenetic analysis

**Figure 7:**
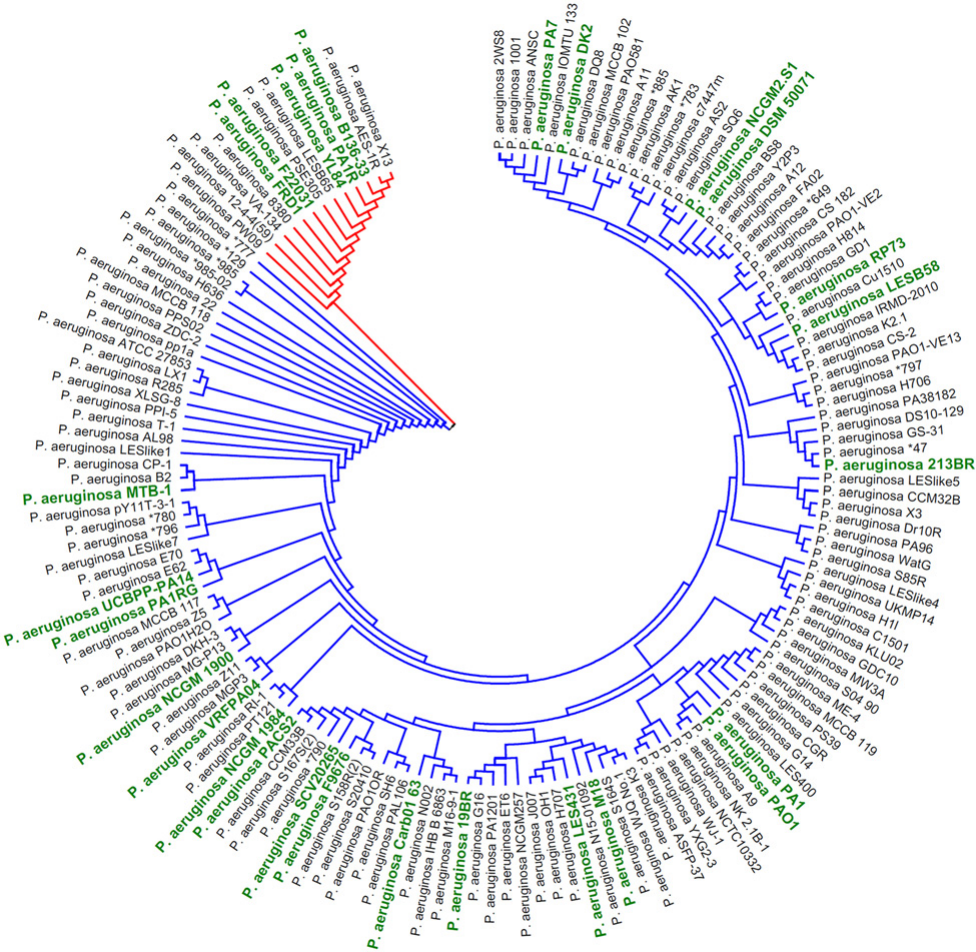
Phylogenetic relationships of *P. aeruginosa* based on 16S rRNA sequences Neighbour-joining method was used to construct a phylogenetic tree. Phylogenetic relationships of 153 *P. aeruginosa* strains (only topology was displayed). Two subgroups are shown in red and blue branches respectively. The 27 compared *P. aeruginosa* strains in Figure 6 are indicated in green and bold font.

The sequences of 16S rRNA are commonly used to establish phylogenetic relationships of bacteria because of their conservative nature and universal distribution [62,63]. A phylogenetic tree was drawn on the basis of 16S rRNA sequences. In *P. aeruginosa* species, the 16S rRNA sequences of different strains slightly differ. This slight difference indicated discriminable evolution processes. The 153 *P. aeruginosa* strains, isolated from different areas around the earth, can be divided into two subgroups (Figure 7). Thus, a distinguishable evolutionary relationship likely exists among them. PA1 is closely related to *P. aeruginosa* PAO1, A9 and NK 2.1B-1. Topology is illustrated in Figure 7 to present the evolutionary relationships among these strains. The original phylogenetic tree is shown with relative genetic distance in Supplementary Figure S1.

#### Pan-genome analysis

**Figure 8:**
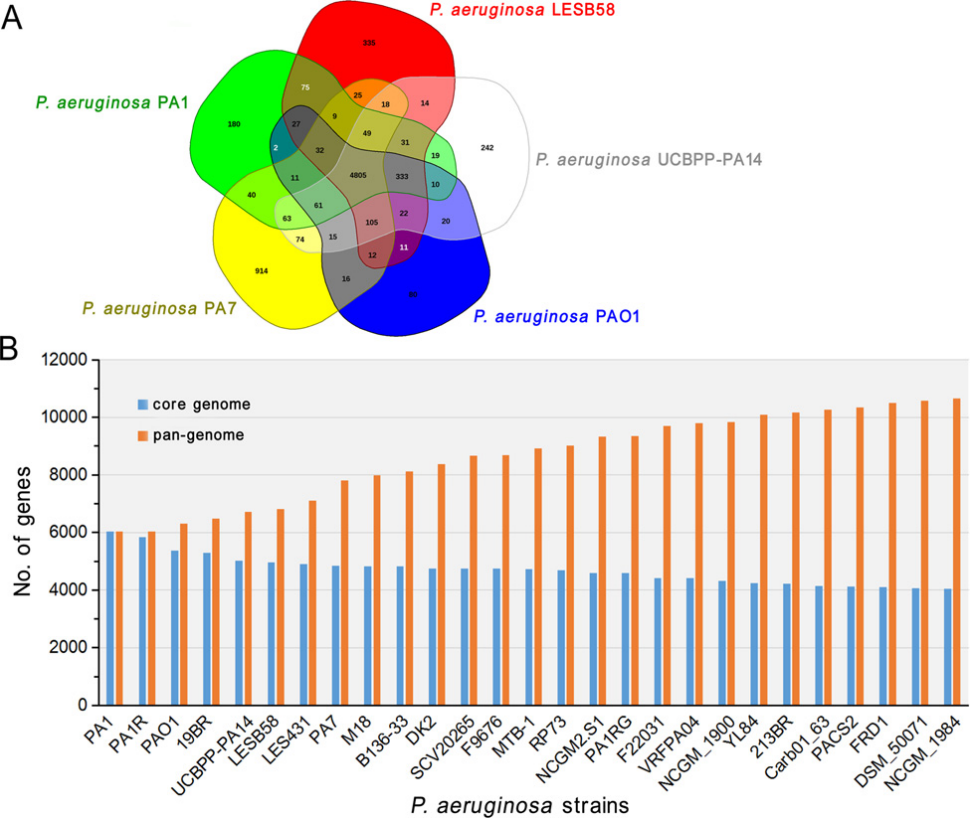
Size of the pan-genome and core genome of *P. aeruginosa*, with the PA1 genome used as a reference (**A**) Venn diagram of five *P. aeruginosa* strains. (**B**) Pan-genome and core genome of the 27 *P. aeruginosa* strains with completed genomes.

The pan-genome of a bacterial species consists of a core genome common to all members of the species and an accessory genome present in at least one member but not in all members of the genus [64]. In the current genomic era, pan-genome analysis helps discover new genes in some species after the genomes of several strains are sequenced; pan-genome analysis also provides evidence supporting minimal genome theory [65]. The Venn diagram illustrates that PA1 shares 4,805 core genes with four other typical *P. aeruginosa* strains, including PAO1, PA7, LESB58 and UCBPP-PA14 (Figure 8A). A total of 2,603 accessory genes are predicted; of these accessory genes, 1,751 are unique, that is, they belong to only one of the five strains. A pan-genomic analysis was performed on the basis of the 27 complete genomes of *P. aeruginosa*. The result demonstrated that the pan-genome of *P. aeruginosa* comprises approximately 4,000 core genes and at least 6,600 accessory genes (Figure 8B). Most of the genes of PA1 within the prophage regions (Figure 2B) and the genomic islands (Figure 5D) are accessory genes of *P. aeruginosa*.

## DISCUSSION

The NGS technologies, such as 454 GS FLX+ (Roche) and Solexa (Illumina), usually produce assembly errors in bacterial genome sequences because of a short read length. For example, sequencing with 454 GS FLX+ (Roche) can only reach a length of 700 bp. Time-consuming and low throughput are shortages of 454 GS FLX+ [66–68]. By contrast, the TGS technologies, such as Pac-Bio SMRT sequencing, can yield long read lengths to enhance *de novo* assembly and enable the direct detection of haplotypes and even whole chromosome phasing [47,48]. Furthermore, SMRT sequencing provides several advantages, including high throughput, fast turnaround time, high consensus accuracy and small amounts of DNA sample [61,69]. Therefore, this technology is very suitable for resequencing projects on bacteria. The new genome sequence of *P. aeruginosa* PA1, resequenced by SMRT technology, is very precise, as confirmed by PCR sequencing. However, the old PA1 genomic sequence, determined using Solexa, contains many errors (Figure 2A). In this sense, the resequencing of the PA1 genome is very necessary and useful to clean the wrong data from outdated techniques or poorly curated datasets.

In the increasingly high-throughput genome sequencing era, a large collection of sequence data have become available in several databases. However, these data mostly display DNA sequences and annotations in a way not easy to read and without an intuitive and visualized genomic analysis. In the present study, several software and online analysis systems were utilized to visualize general genome annotations, the virulence factors, RPs (TFs, TCS and ODP), secretion system proteins, type II T–A pairs and genomic islands of *P. aeruginosa* PA1. Thus, the present study provided a clear presentation of PA1 genomic characteristics useful for further studies.

Elastase, phospholipase or siderophore was not predicted in the PA1 genome (Figure 3). However, this finding does not indicate that PA1 does not encode these virulence factors because current genome annotating methods are unable to precisely predict the functions of all proteins. In the PA1 genome, 1,273 proteins were predicted with unknown functions (Supplementary Table S2). Nevertheless, some proteins previously described as “hypothetical” will be predicted with putative functions as numerous proteins with determined functions have been submitted to public databases. The PA1 genome was predicted to encode 19 T–A pairs (Figure 5C), and one T–A pair is located in an intact prophage region (Figure 2B). The toxin (PA1S_06870) contains a COG2929-like domain, and the antitoxin (PA1S_06865) comprises a RHH-like domain. This T–A system probably contributes to the stability of the prophage.

HGT occurs among bacterial strains; as a result, bacterial virulence changes and genome sequences vary [70]. The main sequence differences of *P. aeruginosa* strains are found in genomic regions contained in mobile genetic elements (MGEs), such as prophages (Figure 2B) and genomic islands (Figure 5D). This finding suggested that they are probably acquired through HGT during bacterial evolution. These distinct regions also indicate the genome plasticity and population structure diversity of *P. aeruginosa* strains [57,71,72]. The 153 *P. aeruginosa* strains could be divided into two subgroups on the basis of the phylogeny analysis of the 16S rRNA sequences (Figure 7). By contrast, whole genome phylogeny analysis with core genome SNPs revealed presumably three groups of *P. aeruginosa* strains [71,73,74]. The third group is represented by PA7. The grouping difference was probably attributed to different genomic sequences used for phylogeny analysis. The core genome of *P. aeruginosa* comprises approximately 4,000 genes (Figure 8B); by comparison, the core genome of *Pseudomonas putida* comprises approximately 3,386 genes [75]. This difference can be explained by their genome sizes because the average length of *P. aeruginosa* genome is close to 6.6 Mbp, but the average length of *P. putida* genome is less than 6.1 Mbp.

As a major nosocomial pathogen, *P. aeruginosa* is responsible for community-acquired infections and is generally associated with contaminated water and solutions. As a versatile opportunistic pathogen, *P. aeruginosa* is intrinsically multidrug-resistant and thus can acquire additional resistances to naturally active antimicrobial agents, such as some β-lactams, aminoglycosides and quinolones. *P. aeruginosa* strains isolated from different niches or clinical origins exhibit variable drug resistance spectra. *P. aeruginosa* PA1 is resistant or intermediate-resistant to 24 antibiotics among the 29 tested antibiotics that include 8 antibiotic categories (Figure 1D). However, the antibiotic resistance mechanisms of *P. aeruginosa* have yet to be fully elucidated [76,77]. Two β-lactam resistance genes, one fosfomycin resistance gene and one amphenicol resistance gene were predicted in the PA1 genome (Supplementary Table S1). In addition, 12 multidrug transporter-related genes were predicted in the PA1 genome, and these genes likely contributed to the multidrug resistance of this bacterium. Novel drug-resistance mechanisms of *P. aeruginosa* should be explored to help control this notorious opportunistic pathogen.

Taken together, the present study describes SMRT resequencing and detailed, visualized, comparative, phylogenetic and pan-genomic analyses of *P. aeruginosa* PA1 strain. We found that the 6,498,072 bp complete genome sequence of PA1, resequenced by using SMRT technology, is relatively accurate, thus cleaning the old and wrong data from outdated sequencing techniques. PA1 exhibits similarity to other *P. aeruginosa* strains but differs in terms of HGT regions, such as prophages and genomic islands. PA1 is closely related to PAO1, and *P. aeruginosa* strains can be divided into two main groups. The pan-genome of *P. aeruginosa* consists of a core genome of approximately 4,000 genes and an accessory genome of at least 6,600 genes. The present study provides a useful basis for future studies of this notorious pathogen.

## Abbreviations

BLAST: Basic Local Alignment Search Tool
BRIG: BLAST Ring Image Generator
CF: cystic fibrosis
CFU: colony forming unit
HGT: horizontal gene transfer
LPS: lipopolysaccharide
MTase: methyltransferase
NGS: next-generation sequencing
ODP: other DNA-binding proteins
PCN: pyocyanin
R–M: restriction–modification
RP: regulatory protein
SMRT: single-molecule real-time
SNP: single nucleotide polymorphism
T–A: toxin–antitoxin
TCS: two-component systems
TF: transcription factor
TGS: third-generation sequencing
